# Emerging approaches for the development of artificial islets

**DOI:** 10.1002/SMMD.20230042

**Published:** 2024-03-07

**Authors:** Jingbo Li, Lingyu Sun, Feika Bian, Stephen J. Pandol, Ling Li

**Affiliations:** ^1^ Department of Endocrinology Zhongda Hospital School of Medicine Southeast University Nanjing China; ^2^ Department of Clinical Laboratory Nanjing Drum Tower Hospital School of Biological Science and Medical Engineering Southeast University Nanjing China; ^3^ Division of Gastroenterology Department of Medicine Cedars‐Sinai Medical Center Los Angeles California USA

**Keywords:** artificial islet, diabetes, hydrogel, microfluidics, regenerative medicine

## Abstract

The islet of Langerhans, functioning as a “mini organ”, plays a vital role in regulating endocrine activities due to its intricate structure. Dysfunction in these islets is closely associated with the development of diabetes mellitus (DM). To offer valuable insights for DM research and treatment, various approaches have been proposed to create artificial islets or islet organoids with high similarity to natural islets, under the collaborative effort of biologists, clinical physicians, and biomedical engineers. This review investigates the design and fabrication of artificial islets considering both biological and tissue engineering aspects. It begins by examining the natural structures and functions of native islets and proceeds to analyze the protocols for generating islets from stem cells. The review also outlines various techniques used in crafting artificial islets, with a specific focus on hydrogel‐based ones. Additionally, it provides a concise overview of the materials and devices employed in the clinical applications of artificial islets. Throughout, the primary goal is to develop artificial islets, thereby bridging the realms of developmental biology, clinical medicine, and tissue engineering.

## Graphical abstract

The islet of Langerhans, functioning as a “mini organ”, plays a vital role in regulating endocrine activities due to its intricate structure. Dysfunction in these islets is closely associated with the development of diabetes mellitus (DM). To offer valuable insights for DM research and treatment, various approaches have been proposed to create artificial islets or islet organoids with high similarity to natural islets, under the collaborative effort of biologists, clinical physicians, and biomedical engineers. This review investigates the design and fabrication of bioartificial islets, considering both biological and tissue engineering aspects. It begins by examining the natural structures and functions of native islets and proceeds to analyze the protocols for generating islets from stem cells. The review also outlines various techniques used in crafting artificial islets, with a specific focus on hydrogel‐based ones. Additionally, it provides a concise overview of the materials and devices employed in the clinical applications of artificial islets. Throughout, the primary goal is to develop artificial islets, thereby bridging the realms of developmental biology, clinical medicine, and tissue engineering.

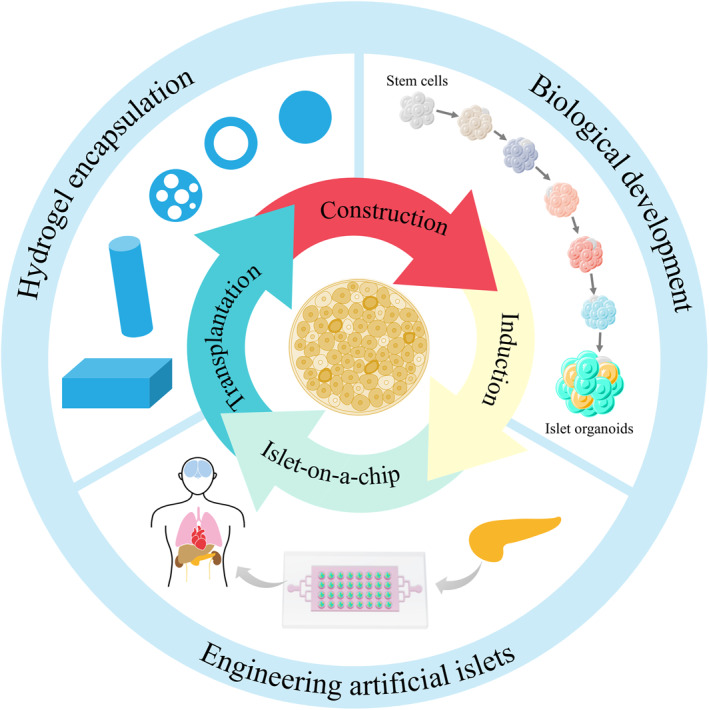

**Key points**

- This review outlined the architecture and biological function of natural islets, as well as the induction from stem cells toward islet organoids.
- The fabrication of artificial islets based on biomedical engineering strategies was systematically illustrated.
- The applications and clinical studies of artificial islets were discussed.
- The progress made in the development of artificial islets has generated optimism for novel approaches in diabetes treatment.

## 1 INTRODUCTION

Islets, organized cellular clusters within the endocrine pancreas, serve a crucial role of secreting hormones.^1^, ^2^ These islets are composed of various cell types, including pancreatic *β* cells, *α* cells, *δ* cells, and PP cells. Each of these cells contributes to maintaining glycemic homeostasis by secreting specific hormones such as insulin, glucagon, somatostatin, and pancreatic polypeptide. When there is insufficient mass or impaired function in these islets, it usually leads to disorders in glycemic control, with the loss of pancreatic *β* cells being a hallmark of diabetes mellitus (DM).^3^, ^4^, ^5^ Individuals with DM experience both acute complications like diabetic ketoacidosis and hyperosmolar hyperglycemic state, as well as chronic complications including diabetic nephropathy, diabetic retinopathy, diabetic peripheral neuropathy, diabetic peripheral vasculopathy, and diabetic foot problems, among others.^6^, ^7^, ^8^ According to the International Diabetes Federation (IDF) Diabetes Atlas, in 2022, an estimated 537 million adults were living with DM, and this number is expected to increase to 783 million by 2045.^9^ As the IDF warns, “Diabetes is spiraling out of control,” thereby imposing a significant medical and financial burden on healthcare systems worldwide.

Artificial islets are engineered structures resembling functional islets or islet organoids created by biological and engineering techniques.^10^, ^11^ Among them, hydrogels are widely employed in constructing artificial islets due to their high biocompatibility and ease of manipulation.^12^, ^13^, ^14^ Based on natural and synthetic hydrogels, both scaffold and non‐scaffold materials have been designed to support the induction and sustained culture of artificial islets.^15^, ^16^, ^17^, ^18^ The incorporation of peptides enhances the chemical and physical properties of hydrogels, opening up numerous possibilities for their application in the fabrication of artificial islets.^19^, ^20^ Moreover, the microfluidic chip integrating islet organoids or islet cell aggregates, often referred to as “islet‐on‐a‐chip”, plays a pivotal role in mimicking the physiological microenvironment for artificial islets.^21^, ^22^, ^23^, ^24^, ^25^ The design of microfluidic chips allows for precise control and real‐time observation of cell cultures, making them ideal platforms for inducing artificial islets in dynamic conditions. Furthermore, researchers have explored the concept of multi‐organ‐on‐a‐chip, which includes investigating crosstalk between islets and the liver, indicating the significant potential of artificial islets in scientific research, as an alternative to animal models or human recipients.^25^, ^26^

In this review, we have summarized the advancements in artificial islets from both biological and engineering perspectives. We commence by outlining the physiological construction and functions of islets in healthy organisms. Subsequently, we delve into the induction procedures for artificial islets, covering both developmental and biological methodologies. In particular, we elaborate on the bioengineering strategies employed in the creation of artificial islets. We also shine a spotlight on the commercialized artificial islets and their potential for clinical applications. Finally, we discuss the existing challenges and the promising future prospects that will guide further research in the development of advanced artificial islets.

## 2 ARCHITECTURE AND FUNCTION OF NATURAL ISLETS

### 2.1 Architecture and cell types of the islet niche

**FIGURE 1:**
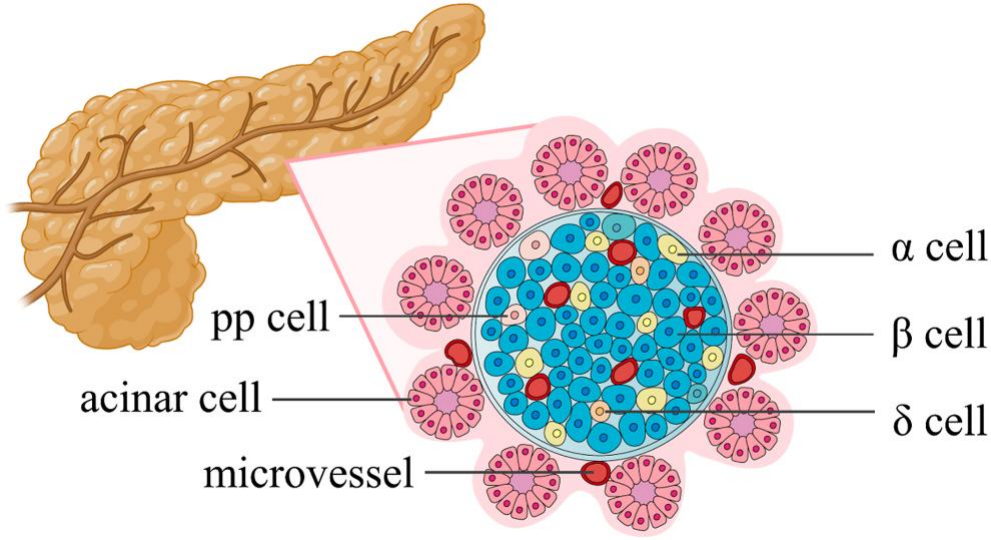
Scheme of pancreas and islet cells. The islet consists of α, β, δ, and pp cells, which are perfused by microvessels and surrounded by the exocrine pancreas. The *α* cells secrete glucagon that elevates the blood glucose. The *β* cells secrete insulin, which is a unique hormone for reducing hyperglycemia. The *δ* cells secrete somatostatin, a hormone with various effects on countering other hormones, such as growth hormone, thyroid‐stimulating hormone, insulin, and glucagon. The pp cells secrete pancreatic polypeptide, regulating the metabolic behaviors of human bodies via inhibiting the release of cholecystokinin and pancreatic enzymes. Microvessels penetrate and accompany the islets, transporting the necessary nutrients and secreted hormones. Around the endocrine pancreas, the islets, spread the exocrine pancreas consisting of the acinar and ducts. The acinar cells secrete digestive enzymes including *α*‐amylase, lipase and proteases, which are responsible for the hydrolysis of carbohydrates, fats and proteins, respectively.

The islets of Langerhans, also referred to as the endocrine pancreas, are meticulously organized micro‐organs with an average diameter of approximately 100 μm.^1^ These human islets are enveloped by a double basement membrane, effectively separating them from the exocrine pancreas, including pancreatic ducts and acini. Within these islets, there exists a diverse population of endocrine cells: *α* cells (15%–20%), *β* cells (70%–80%), *δ* cells (5%–10%), pancreatic polypeptide (PP) cells (5%–10%), and ε cells (around 1%). Notably, these endocrine cells with distinctive roles are arranged within the islets in compartmental proportions, as depicted in Figure 1.^27^, ^28^, ^29^ In addition to endocrine cells, immune cells such as macrophages, dendritic cells, and T cells fulfill important roles in immune regulation within the islets. The islets are also characterized by a dense network of microvessels responsible for facilitating the transport of biochemical molecules.^30^, ^31^ In the interstitial spaces between blood vessels and endocrine cells, nerve fibers are found to contribute to the intricate neural control of islet function. Furthermore, the extracellular matrix (ECM) of the islets primarily comprises laminin, collagen IV, and collagen VI, providing structural support and signaling cues within the islets.^19^, ^32^, ^33^

### 2.2 Biological functions of islets

As intricate “tiny organs”, islet function as integrated units housing a collection of endocrine cells and a network of circulatory and nervous structures that are vital for metabolic regulation.^34^, ^35^, ^36^ Endocrine cells within the islets secrete hormones designed to either raise or lower blood glucose levels depending on the specific cell types. One of the pivotal hormones produced is insulin, a protein hormone primarily secreted by the pancreatic islet *β* cells. Insulin plays a critical role in the regulation of glucose metabolism and the maintenance of glucose homeostasis making it a cornerstone in the treatment of diabetes.^37^, ^38^, ^39^, ^40^ Insulin's primary physiological effect revolves around metabolic control. It facilitates glucose uptake and utilization by various tissues and cells, ultimately promoting glycogen synthesis while inhibiting gluconeogenesis, consequently reducing blood glucose levels. In the context of adipose metabolism, insulin also aids in fatty acid synthesis and storage while concurrently restraining lipolysis.^41^, ^42^, ^43^ Furthermore, insulin significantly enhances protein synthesis, impacting the intracellular transport of amino acids. Notably, insulin stands as the sole hormone capable of decreasing blood glucose levels, making the assessment of insulin content a crucial aspect in understanding metabolic diseases related to insulin.^44^, ^45^ In contrast, glucagon, another hormone originating from the islets and synthesized by *α* cells, functions as an antagonist in blood glucose regulation. Elevated levels of glucagon stimulate the synthesis and secretion of insulin.^46^, ^47^ It is important to note that under conditions of hyperinsulinemia, the secretion of glucagon by *α* cells is inhibited.

Somatostatin (SST), secreted by *δ* cells, plays a regulatory role by inhibiting the secretion of various hormones, including growth hormone, thyroid‐stimulating hormone, insulin, and glucagon. SST also influences the absorption and nutritional functions of the gastrointestinal tract.^48^ Pancreatic polypeptide (PPY), secreted by PP cells, exerts its influence by inhibiting the release of cholecystokinin and pancreatic enzymes, which is contingent on dietary components such as protein, adipose tissue, and carbohydrates.^49^ In contrast, ε cells, a smaller subgroup of islet cells, secrete ghrelin, a hormone with opposing effects to insulin. Ghrelin may play an important role in the development and differentiation of islets.^50^ In addition to these endocrine cells, the vascular network within the islets provides immediate glucose responsiveness, enabling the detection of fluctuations in blood glucose levels and facilitating the secretion of insulin or glucagon. Furthermore, nerve fibers actively participate in the regulation of hormone secretion through the nervous system.

## 3 INDUCING ISLETS BY DEVELOPMENTAL AND BIOLOGICAL STRATEGIES

### 3.1 Generation of stem cell‐derived insulin‐secreting cells

**FIGURE 2:**
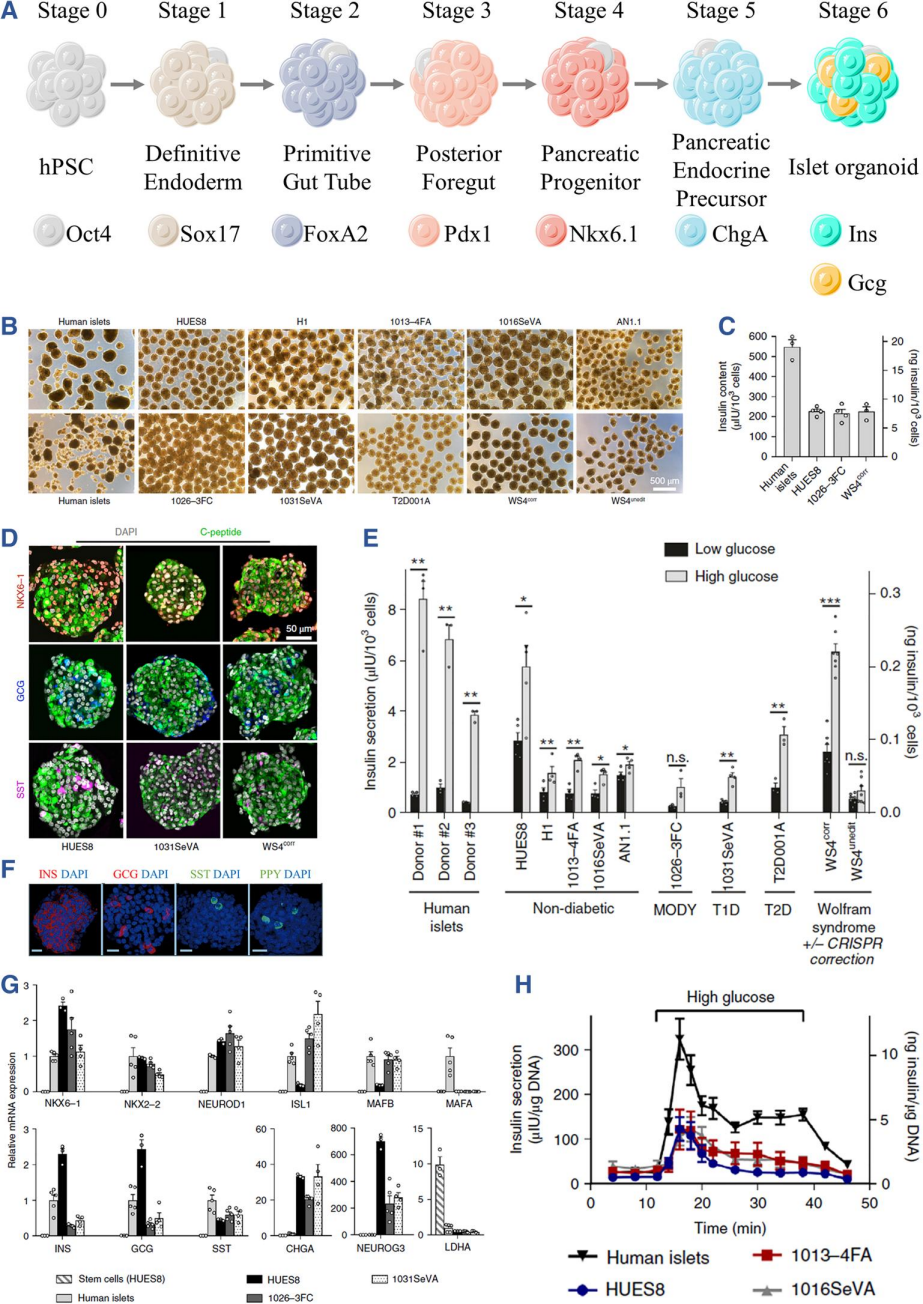
Differentiation of stem cells toward islet organoids. (A) Biomarkers for each stage are shown. (B) Representative images of the induced islet organoids from various hPSC cell lines. (C) Insulin contents of islet organoids derived from various hPSC cell lines. (D) Immunofluorescent images of islet organoids for C‐peptide, NKX6.1, glucagon (GCG), and somatostatin (SST). (E) Glucose responsiveness of islet organoids derived from hPSC under different genetic backgrounds. *Source*: Reproduced with permission.^54^ Copyright 2021, The Authors, published by Springer Nature. (F) Immunofluorescent images of islet organoids for insulin (Ins), GCG, SST, and polypeptide (PPY). *Source*: Reproduced with permission.^55^ Copyright 2022, Springer Nature. (G) Relative mRNA expression of islet genes in hPSC‐derived islet organoids and primary human islets. (H) Insulin secretion of islet organoids on dynamic GSIS assay. *Source*: Reproduced with permission.^54^ Copyright 2021, The Authors, published by Springer Nature.

Pancreatic *β* cell‐like cells or stem cell‐derived *β* cells (SC‐β) with insulin‐secreting functionality are generated from human embryonic stem cells (hESCs) or induced pluripotent stem cells (iPSCs) through a six‐stage oriented differentiation process (Figure 2A).^51^, ^52^, ^53^ The induction of SC‐β cells begins at stage 1, where stem cells are directed toward the formation of definitive endoderm (DE). Subsequently, at stage 2, the sequential development of the primitive gut tube (PGT) takes place, followed by the posterior foregut (PFG) at stage 3. The next stage, stage 4, leads to the induction of pancreatic progenitors (PP) with the potential for pancreas differentiation. At stage 5, these PP cells give rise to pancreatic endocrine precursors (PEP). Finally, at stage 6, the SC‐β cell, also referred to as SC‐islet, is achieved, displaying the ability to secrete hormones like insulin.

According to the protocols proposed by Millman et al., SC‐β cells can be derived from various stem cell lines, exhibiting insulin‐secreting function similar to human islets (Figure 2B,C).^54^ These SC‐β cells also express biomarkers of islets, such as C‐peptide or insulin, glucagon, SST, and PPY (Figure 2D,F).^54^, ^55^ Notably, stem cells derived from specific genetic background affects the destination of SC‐β cells, consistent with their original donors (Figure 2E). As the cells differentiate into the mature SC‐β cells, the potency of stem cells is inhibited while the genetic expression related to human islets becomes eminent (Figure 2G). Specifically, the glucose responsiveness demonstrates a milestone of SC‐β maturation, when the cells temporally secrete insulin under the stimuli (Figure 2H).^54^

In 2007, Deng et al. introduced a method for inducing insulin‐producing cells derived from hESCs using chemical molecules. In their study, Activin A was employed in stage 1 for the formation of DE, while all‐trans retinoic acid facilitated pancreatic differentiation. Additionally, basic fibroblast growth factor (bFGF) and nicotinamide were utilized to promote the maturation of SC‐β cells. Upon transplantation into the renal capsules of streptozotocin (STZ)‐induced diabetic mice, these SC‐β cells demonstrated islet‐like functionality with insulin‐secreting capacity, resulting in sustained euglycemia.^56^ Through further refinements of this protocol, SC‐β cells induced by small chemical molecules have found broad applications in biological research.

In the quest for adult stem cells within pancreatic islets, the research group led by Zeng discovered a population of Procr‐positive (Procr+) cells in mouse islets through single‐cell sequencing (scRNA‐seq). Procr is a surface protein previously identified as a marker for stem cells in various adult tissues, including the mammary gland, endothelium, and hematopoietic system. The Procr + cells within mouse islets exhibited characteristics suggestive of epithelial‐mesenchymal transition. To investigate these cells in vivo, they developed Procr reporter gene mice (Procr‐mGFP‐2A‐LacZ). Remarkably, these Procr + cells lacked the biomarkers typically associated with differentiated islet cells, indicating an undifferentiated state. In vivo lineage tracing experiments (Procr‐CreERT2, Rosa26‐confetti) revealed that this Procr + cell population could differentiate into all islet cell types (α, β, δ, PP cells) under normal physiological conditions. These experiments provided compelling evidence that Procr + cells serve as adult stem cells within the islets.^55^, ^57^

The researchers further established an in vitro 3D culture system in which Procr + islet stem cells were co‐cultured with vascular cells to create functional islet organoids, encompassing all cell types found in pancreatic islets. These organoids exhibited remarkable similarity to native mouse islets in terms of function, morphology, ultrastructure, and transcriptome. They responded rapidly to glucose stimulation and secreted insulin. Furthermore, they could be cultured and passaged in vitro for over 20 generations. When transplanted into diabetic mouse models, these pancreatic islet organoids significantly improved the blood glucose levels, underscoring their therapeutic potential. These in vitro culture methods not only highlight the application potential of Procr + cells but also reaffirm their status as stem cells within the islets.

### 3.2 Identification of the induction of islet organoids

#### 3.2.1 Biomarkers of differentiating cells at sequential stages

**FIGURE 3:**
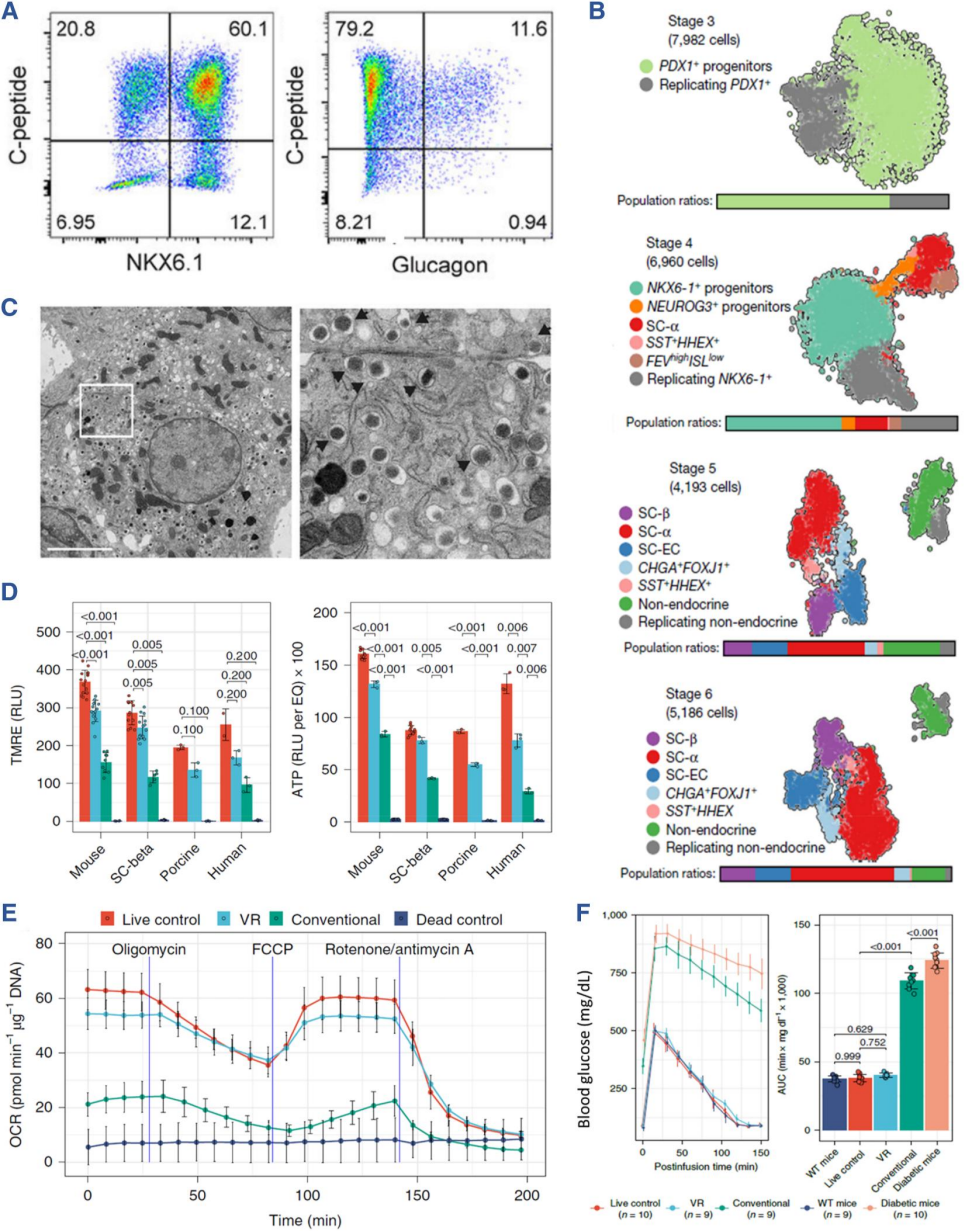
Identification of SC‐β and islet organoids. (A) Representative flow cytometric dot plots of dispersed stage 6 SC‐β cells immune‐stained for the indicated markers. *Source*: Reproduced with permission.^58^ Copyright 2021, The Authors, published by American Association for the Advancement of Science. (B) Single‐cell RNA sequencing of in vitro *β*‐cell differentiation. *Source*: Reproduced with permission.^59^ Copyright 2019, The Authors, published by Springer Nature. (C) Insulin granules observed via TEM. *Source*: Reproduced with permission.^63^ Copyright 2021, the American Diabetes Association. (D) The mitochondrial membrane potential evaluated by TMRE. (E) Typical OCR curve of islets. *Source*: Reproduced under terms of the CC‐BY license.^62^ Copyright 2022, The Authors, published by Springer Nature. (F) Glucose tolerance evaluated by IPGTT. *Source*: Reproduced under terms of the CC‐BY license.^62^ Copyright 2022, The Authors, published by Springer Nature.

Differentiated cells at specific stages of the differentiation process are characterized by the expression of corresponding biomarkers. In classical studies, the identification of these biomarkers has traditionally relied on techniques such as flow cytometry and immunohistochemistry to assess their expression and proportion (Figure 2D,F; Figure 3A).^54^, ^55^, ^58^ However, more recent approaches have incorporated transcriptome and genetic analysis to comprehensively evaluate stem cell‐derived cells (Figure 3B).^59^, ^60^, ^61^

To outline the progression of differentiation, several key biomarkers are observed at different stages. Notably, at stage 1, there is an elevation in the expression of Oct‐4 and SOX17. As the differentiation process continues, SOX17 expression continues to increase, and the expression of HNF‐1B also becomes evident into stage 2. Stage 3 sees the emergence of PDX1 in conjunction with the already elevated HNF‐1B, signifying the formation of what is known as the pancreatic foregut tube (PFT). Subsequently, at stage 4, the expression of the pancreatic lineage marker NKX6.1 becomes prominent. As differentiation progresses from stage 4 to stage 6, there is a notable expression of PDX1, chromogranin, and insulin. This expression pattern suggests the maturation of pancreatic progenitors (PP) at stage 4 followed by pancreatic endocrine precursors (PEP) and, ultimately, SC‐β at stages 5 and 6, respectively.^53^, ^59^

#### 3.2.2 Functional evaluation of stem cell‐derived islets

In the characterization of SC‐β cells, it is crucial to assess both their morphology and function. To begin with, SC‐β cells are observed under bright field microscopy to evaluate their morphological characteristics. In addition, the organelles within SC‐β cells, particularly insulin granules, can be investigated using transmission electron microscopy (TEM) (Figure 3C).^63^ Furthermore, the viability of SC‐β cells can be examined through various assays, such as Calcein am/propidium iodide (PI) staining or Cell‐Counting‐Kit‐8. To detect cell death, TdT‐mediated dUTP nick end labeling (TUNEL) staining can be employed to identify DNA fragmentation indicative of necrosis. Additionally, the detection of Annexin V translocation serves as a positive indicator of apoptosis.

Functional evaluation of SC‐β cells encompasses their glucose responsiveness and metabolic functionality. As cells are responsible for hormone secretion, the primary function of SC‐β cells is to secrete insulin in response to elevated glucose levels. This in vitro insulin secretion can be assessed using glucose‐stimulated insulin secretion (GSIS) and potassium‐stimulated insulin secretion (KSIS) assays. In these tests, SC‐β cells are initially exposed to low‐glucose agents, followed by stimulation with high glucose or potassium ion stimuli (Figure 2E,H). The quantity of insulin secreted at each phase is recorded and calculated, yielding the stimulation index (SI), which indicates the fold increase in secreted insulin compared to the basal value.

Beyond insulin secretion, the metabolic status of SC‐β cells offers valuable insights into their activity. The mitochondrial membrane potential can be estimated using tetramethylrhodamine ethyl ester (TMRE) (Figure 3D). Additionally, mitochondrial oxidative phosphorylation can be assessed through the measurement of the oxygen consumption rate (OCR). Generally, basal mitochondrial respiration is measured under normal conditions. Subsequently, the addition of oligomycin inhibits ATP synthase, resulting in a significant decrease in OCR (reflecting ATP production) while proton leakage remains. Following stimulation with carbonyl cyanide 4‐trifluorometheoxyphenylhydrazone (FCCP), an uncoupler of oxidative phosphorylation, OCR sharply rises to reach its peak (maximal respiration). The difference between maximal and basal respiration is termed the spare respiratory capacity. Furthermore, electron transfer inhibitors like antimycin A can be employed to completely inhibit electron transfer and thus minimize OCR (Figure 3E).

An advanced method for evaluating the functionality of SC‐β cells is to transplant these insulin‐secreting cells into diabetic animal models. The traditional transplantation strategy involves renal subcapsular transplantation, offering partial immune isolation effects and minimally invasive procedures. As the study develops in depth, alternative graft sites have been explored, including the omentum, adipose tissue, muscle, and subcutaneous tissues, all showing potential for islet cell transplantation. Following SC‐β cell transplantation, continuous monitoring of blood glucose levels and body weights provides essential data on the effects of the grafts on diabetic models. For the in vivo functional evaluation of SC‐β cells, the intraperitoneal glucose tolerance test (IPGTT) serves as a valuable indicator of glucose responsiveness in diabetic animals. The concentrations of insulin and C‐peptide along with blood glucose levels at various time points are carefully examined and compared between the experimental and control groups. The area under the curve (AUC) of the IPGTT further illustrates the capacity of transplanted SC‐β cells to reduce blood glucose levels (Figure 3F).^62^

## 4 STRATEGIES FOR ENGINEERING ARTIFICIAL ISLETS

### 4.1 Hydrogels in fabricating artificial islets

**FIGURE 4:**
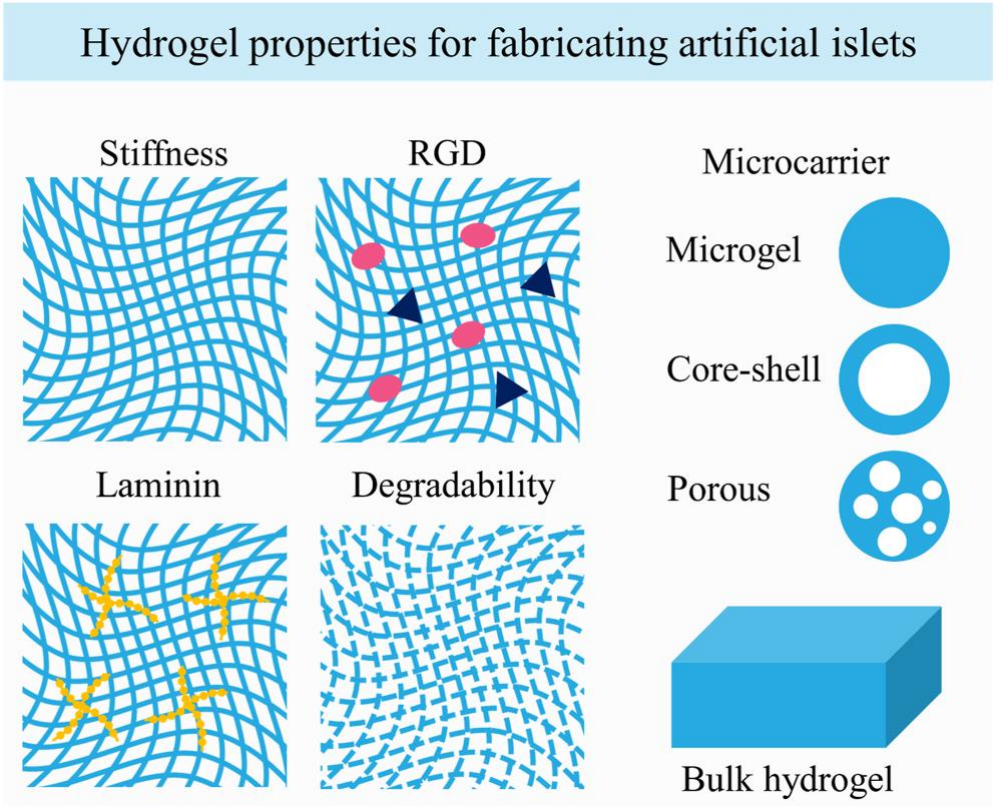
Physiochemical and biological properties of hydrogels for the fabrication of artificial islets.

Hydrogels are 3D biomaterials capable of absorbing substantial quantities of water while retaining their structural integrity. Hydrogels play a pivotal role in the field of tissue engineering, catering to specific requirements in a variety of conditions.^64^, ^65^, ^66^, ^67^, ^68^ When it comes to the induction of islet organoids, hydrogels serve as a crucial supporting matrix both within scaffold and non‐scaffold structures.^69^, ^70^ Moreover, hydrogels address several critical challenges encountered in islet transplantation, including issues related to host immune response, oxygen supply, and the establishment of adequate vasculature.^71^, ^72^ The development of artificial islets benefits significantly from the utilization of hydrogels, combining the intrinsic biological, physical, and chemical properties of both natural and synthetic hydrogels (Table 1). The functional attributes of hydrogels are intricately influenced by several key factors. These factors encompass the mechanical rigidity of the hydrogel, peptide modifications, including laminin and bioactive sequences such as arginine‐glycine‐aspartate (RGD) as well as the hydrogel's degradability. Moreover, the morphological characteristics of hydrogels play an important role in governing molecular permeation and transplantation protocols. The inherent controllability of hydrogels facilitates their deliberate engineering into various forms ranging from microcarriers like microspheres and microfibers to bulk structures, thereby affording researchers a versatile strategy for biomedical applications (Figure 4). This fusion of hydrogel properties empowers the field of artificial islets to advance and meet the complex demands of islet transplantation and regenerative medicine.

**TABLE 1:** Engineering artificial islets based on hydrogels.

Hydrogel	Modifications	Mechanism/biological function	References
Alginate	RGD, co‐culture with MSC	Enhance cell survival	Laporte et al.^73^
	RGD	Reduce fibrosis	Bochenek et al.^74^
	Sulfobetaine	Reduce fibrosis	Liu et al.^75^
	PEO, CMC	Ameliorate high blood glucose	Liu et al.^76^
Chitosan	Alginate	High uniformity, biocompatibility, stability, and permeability	Liu et al.^77^
	Alginate, PEG	Inhibit IL‐2 secretion	Najafikhah et al.^78^
	Alginate/HA	Prevent the infiltration of NK cells	Kim et al.^79^
Agarose	SEK‐1005	Facilitate angiogenesis	Kuwabara et al.^80^
	Co‐culture with MSC	Immune protection	Nakafusa et al.^81^
HA	PEGDA, core‐shell spherification	Nontoxic microencapsulation	Harrington et al.^82^
GelMA	PEO, co‐culture with MSC	Immune modulation	Sun et al.^83^
PEG	Chondroitin sulfate	Immune protection	Yang et al.^84^
	PEG‐b‐PLA nanoencapsulation	Reduces cell membrane damage	Kim et al.^85^
	GelMA	Regulate microenvironment	Li et al.^86^
	VEGF	Facilitate angiogenesis	Scheiner et al.^87^
	PD‐L1	Immune modulation	Coronel et al.^88^

#### 4.1.1 Natural hydrogel‐based platforms

**FIGURE 5:**
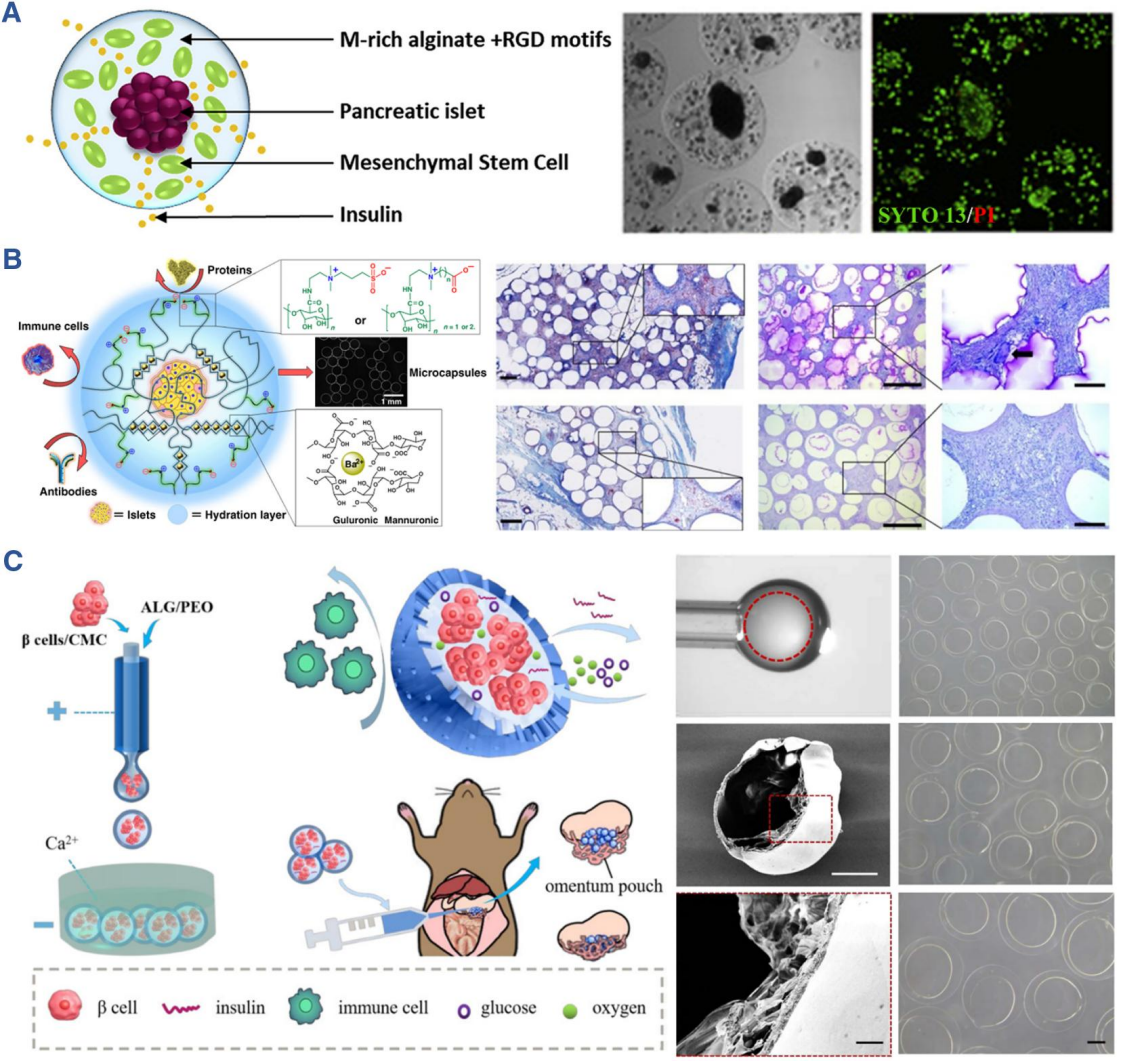
Alginate hydrogels for developing artificial islets. (A) Alginate hydrogel composite RGD motifs for pancreatic islet encapsulation. *Source*: Reproduced with permission.^73^ Copyright 2020, Elsevier B.V. (B) Design of zwitterionically modified alginates and their effects on reducing fibrosis. *Source*: Reproduced under terms of the CC‐BY license.^75^ Copyright 2019, The Authors, published by Springer Nature. (C) Core‐shell structure islet cell encapsulation based on microfluidic electrospray technology. *Source*: Reproduced under terms of the CC‐BY license.^76^ Copyright 2022, The Authors, published by Springer Nature.

Natural polymers with high biocompatibility, abundant source, and maneuverability are beneficial for prompting the induction of islet organoids, such as alginate, chitosan, agarose, hyaluronic acid (HA), and silk. By controlling chemical and physical properties of these biomaterials, the stiffness, the modifications, the integration of peptides, as well as the degradation rate, can be tailored for engineering artificial islets. Sodium alginate consists of 1,4‐linked‐D‐mannuronic acid and L‐guluronate residues, whose mechanical strength or viscosity is proportional to the percentage of guluronic acid, and inversely proportional to that of mannuronic acid. By designing the morphology of hydrogels, alginate hydrogel loading with islet cells was achieved, demonstrating fiber, microcapsule, and macrocapsule structures. Besides, the modification of alginate endows the hydrogel with higher affinity to cell survival. Co‐culture of mesenchymal stem cells (MSCs) and islets in RGD alginate achieved higher cell viability and VEGF secretion (Figure 5A).^73^ Similarly, manipulating the microenvironment around stem cell‐derived *β* cells via RGD alginate enhanced the formulation of *β* cellular spheroids and insulin secretion. A study conducted in non‐human primate models suggested that the alginate formulation, Z1‐Y15, the most suitable for reducing fibrosis.^74^ Moreover, Liu et al. employed sulfobetaine to modify alginate for encapsulating rat islets. The hydrogel with zwitterionic groups functioned in ameliorating fibrosis and cellular overgrowth around the graft site when transplanted into mice, dogs, and pigs (Figure 5B).^75^ Liu and colleagues harnessed the advantages of microfluidic electrospray technology to craft core‐shell microcapsules containing pancreatic *β* cells from the INS‐1 cell line. They achieved this by dispersing the INS‐1 cells within a carboxymethyl cellulose (CMC) solution, effectively encapsulating them within hydrogel shells composed of alginate. These microcapsules were subsequently transplanted into the omentum of diabetic mice, demonstrating significant therapeutic effects in alleviating hyperglycemia (Figure 5C).^76^

**FIGURE 6:**
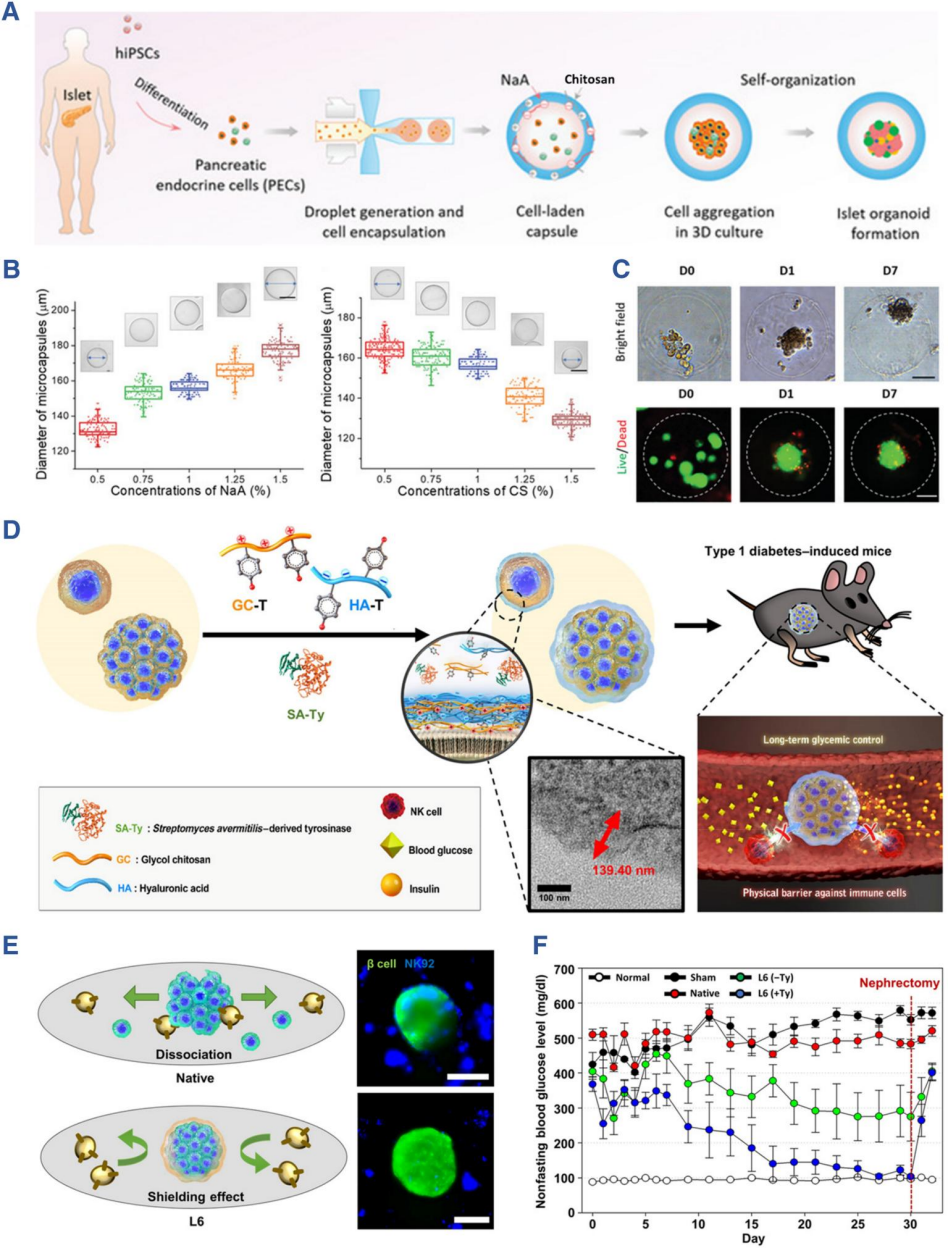
Chitosan for developing artificial islets. (A) All‐in‐water microfluidic system for fabricating binary capsules based on alginate and chitosan. (B) Relationship between hydrogel concentration and diameters of capsules. (C) Bright field and live/dead images of cells encapsulated in the capsules during the culture. *Source*: Reproduced under terms of the CC‐BY license.^77^ Copyright 2020, The Authors, published by John Wiley and Sons. (D) The enzymatic cross‐linking‐based hydrogel nanofilm caging system on pancreatic *β* cell spheroid prevented the infiltration of NK cells. (E) Illustrative and immunofluorescence images of the cells with/without the infiltration of NK cells. (F) Blood glucose levels of diabetic mice after pancreatic *β* cell transplantation. *Source*: Reproduced with permission.^79^ Copyright 2021, The Authors, published by American Association for the Advancement of Science.

As a linear polysaccharide, chitosan is made of *β*‐(1→4)‐linked D‐glucosamine and N‐acetyl‐D‐glucosamine.^89^ Specifically, chitosan contributes to the suppression of inflammatory cytokines, including TNF‐α, IL‐6, IL‐4 receptors, and the proliferation of T cells. Usually, chitosan is employed as composite biomaterials for fabricating the artificial islets rather than encapsulating cells directly. Liu et al. proposed an all‐in‐water microfluidic system for fabricating binary capsules based on the oppositely charged alginate and chitosan. They produced the artificial islets with stem cell‐derived islets, which demonstrated high uniformity, biocompatibility, stability, as well as permeability (Figure 6A–C).^77^ Additionally, Najafikhah et al. produced a device consisting of chitosan, alginate, and polyethylene glycol (PEG) layers to inhibit IL‐2 secretion.^78^ In another work, a delivery device designed by Kim et al. composited chitosan with alginate or HA prevented the infiltration of NK cells (Figure 6D–F).^79^

**FIGURE 7:**
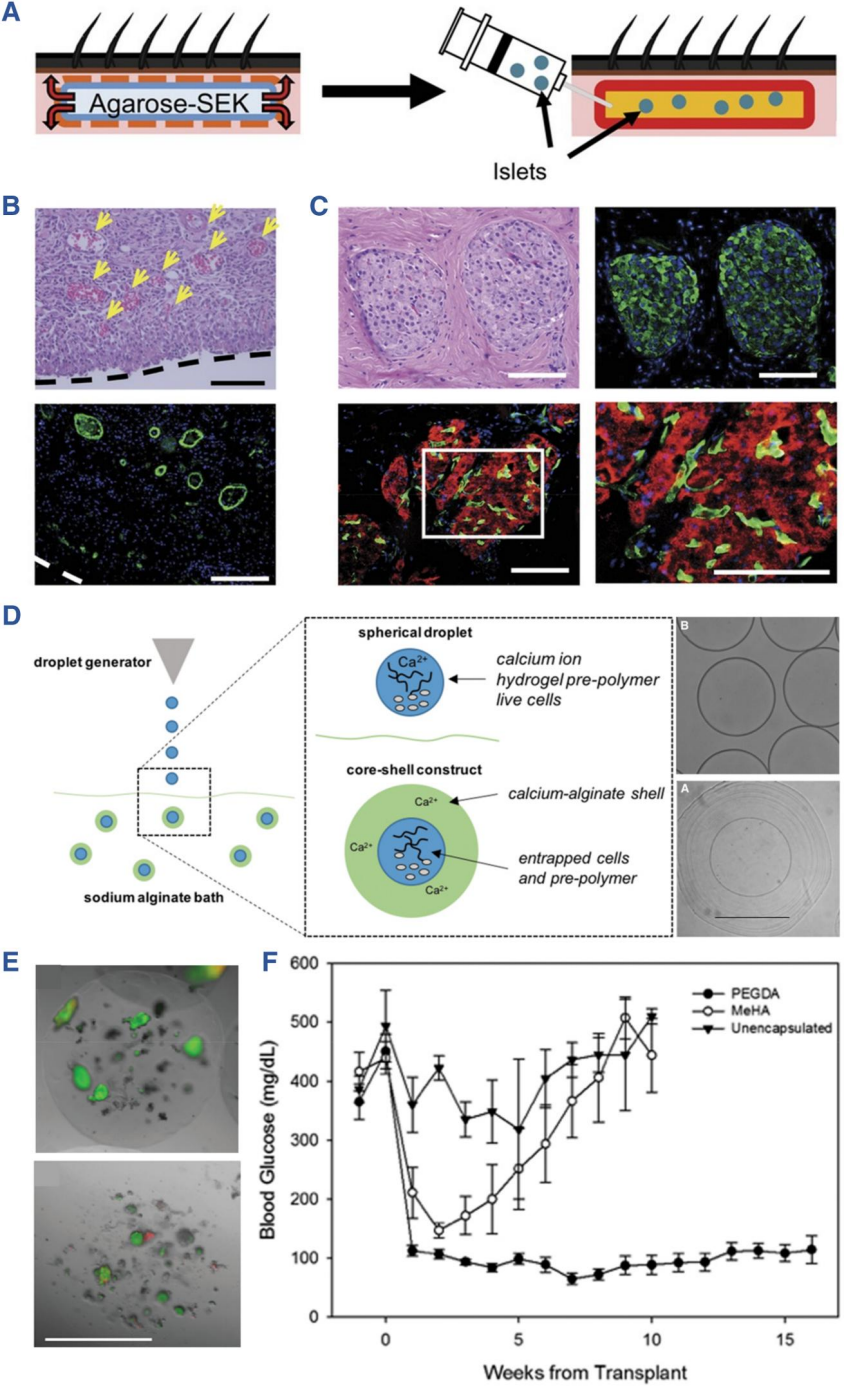
Agarose and hyaluronic acid for developing artificial islets. (A) SEK‐1005 with agarose‐SEK rods to pre‐vascularize a subcutaneous site for allogeneic islet transplantation without immunosuppression. (B) Histological images indicate microvessels at the graft site. (C) Immunofluorescence images of transplanted islets. *Source*: Reproduced with permission.^80^ Copyright 2018, Wolters Kluwer Health, Inc. (D) Schematic of core‐shell spherification method based on hyaluronic acid and polyethylene glycol diacrylate. (E) Live/dead images of the fabricated microspheres. (F) Blood glucose levels of mice transplanted with the microspheres. *Source*: Reproduced under terms of the CC‐BY license.^82^ Copyright 2021, The Authors, published by Mary Ann Liebert, Inc.

Agarose has been used as a microencapsulation hydrogel for islet transplantation since 1987.^80^ A cyclic oligopeptide, SEK‐1005, was combined with agarose rod, leading to improved pre‐vascularization of islet transplantation. It was used for transplanting islets into the subcutaneous space without immunosuppression, which facilitated angiogenesis at the graft site. Additionally, immunofluorescence images suggested the expression of insulin and glucagon in transplanted islets (Figure 7A–C).^80^ In a recent study conducted by Nakafusa et al., co‐transplantation of MSCs with agarose rods enhanced the transplantation of BALB/c islets without immunosuppression at the inguinal subcutaneous white adipose tissue (ISWAT). This study indicated that prevention of islet allograft rejection without immunosuppression is feasible with the use of syngeneic TGF‐β–producing MSCs expanded in the ISWAT after the treatment with bFGF, providing a novel strategy for prevention of islet allograft rejection without immunosuppression.^81^

HA resides in natural extracellular matrix of living bodies. It is a non‐sulfated glycosaminoglycan applied to clinical situations such as wound repair, cosmetology, and ophthalmic operations. HA holds the capacity of anti‐inflammatory owing to its effects on CD44 receptors or CD168.^90^ Harrington et al. fabricated a core‐shell microencapsulation device for islet transplantation using HA hydrogel. It restored normoglycemia in diabetic mice by 4 weeks (Figure 7D–F).^82^ Considering the broad applications in other fields, HA could be an alternative biomaterial for islet transplantation.

#### 4.1.2 Synthetic hydrogel‐based platforms

**FIGURE 8:**
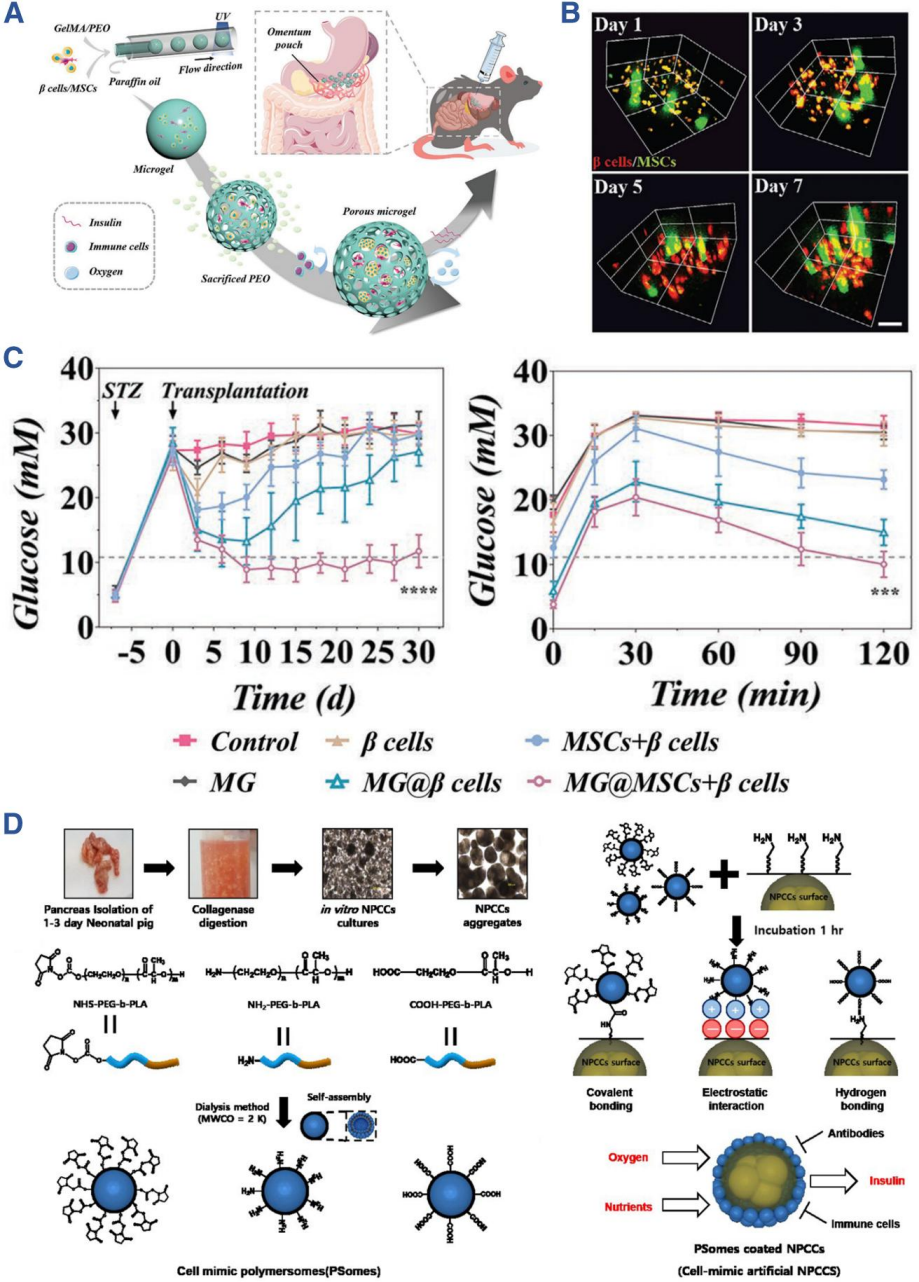
GelMA and PEG for developing artificial islets. (A) Porous microgels designed for the transplantation of *β* cells. (B) Confocal images of the porous microgels. (C) Blood glucose observation and IPGTT results in diabetic mice treated with microgels. *Source*: Reproduced with permission.^83^ Copyright 2023, John Wiley and Sons. (D) Synthesis scheme and structures of cell‐mimic polymersome (PSome)‐shielded islets for long‐term immune protection of neonatal porcine islet‐like cell clusters (NPCCs). *Source*: Reproduced with permission.^85^ Copyright 2020, Royal Society of Chemistry.

GelMA hydrogels exhibit characteristics reminiscent of the natural extracellular matrix (ECM) because they contain peptide motifs that enable cell attachment and responsiveness to matrix metalloproteinase, facilitating cell proliferation and spreading within GelMA‐based scaffolds. Sun and his colleagues embarked on the development of gelatin methacrylate (GelMA)/poly (ethylene oxide) (PEO) porous microgels (MGs) designed for the transplantation of *β* cells. In this innovative approach, pancreatic *β* cells were embedded within the GelMA hydrogel through a photo‐polymerization process, while PEO was selectively sacrificed to create a porous structural framework. For the transplantation procedure, MSCs were strategically co‐cultured to play a pivotal role in immune modulation. This co‐cultivation not only facilitated the engraftment of the microgels but also enhanced the functionality of insulin secretion, showcasing the promising potential of this approach for diabetes treatment (Figure 8A–C).^83^

**FIGURE 9:**
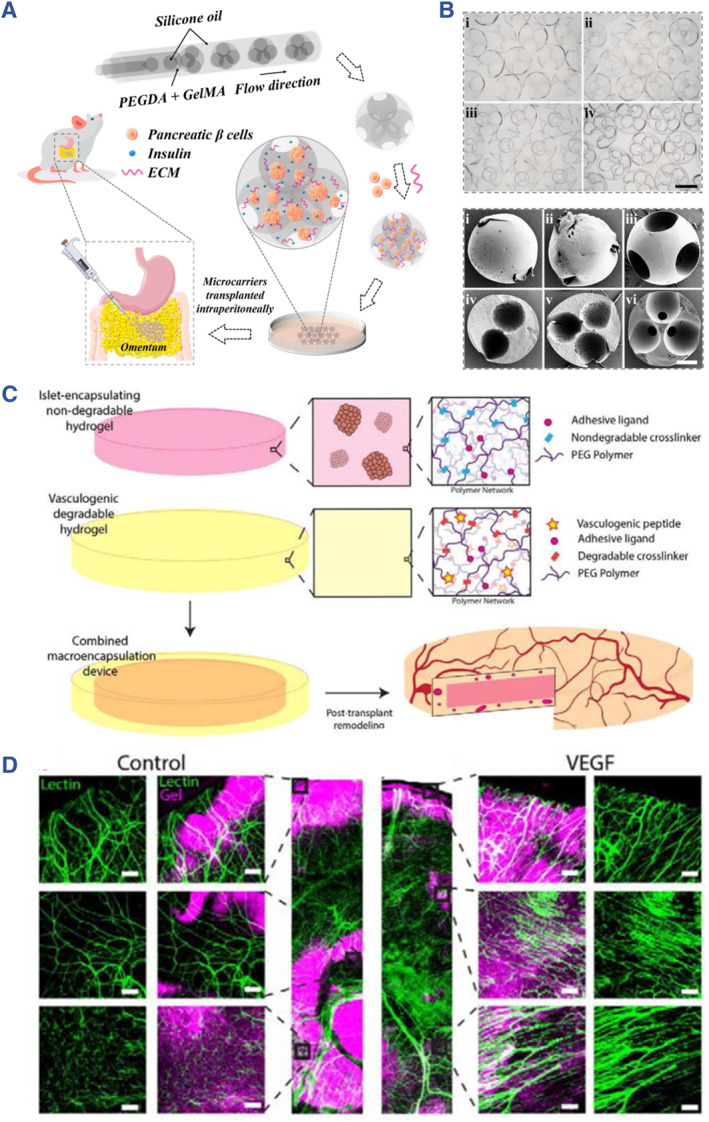
PEG for developing artificial islets. (A) Porous hydrogel microcarriers loading with pancreatic *β* cell aggregates using a microfluidic double emulsion strategy. (B) Bright field and SEM images of the porous microcarriers. *Source*: Reproduced with permission.^86^ Copyright 2022, Elsevier B.V. (C) The vascularized synthetic PEG macro‐encapsulation device for islet transplantation. (D) Confocal images of the hydrogel with and without VEGF. *Source*: Reproduced with permission.^91^ Copyright 2018, Elsevier B.V.

PEG and PEG‐modified (pegylation) are employed in artificial islet systems for ameliorating immune responses as well as facilitating islet functions.^91^ According to Yang et al., transplanted islets could be protected by a nano‐coated PEG consisting of chondroitin sulfate from host immune attack.^84^ Kim et al. developed novel nanoencapsulation of neonatal porcine islet‐like cell clusters (NPCCs) with cell‐mimic polymersomes (PSomes) based on PEG‐b‐PLA (poly(ethylene glycol)‐b‐poly(DL‐lactic acid)) for maintaining transplant survival (Figure 8D).^85^ Another innovative approach to support the culture of pancreatic *β* cells involved the creation of microcarriers as scaffolds. Li and colleagues devised porous hydrogel microcarriers that housed pancreatic *β* cell aggregates utilizing a microfluidic double emulsion strategy. These microcarriers harnessed the cell affinity of GelMA and the non‐adhesiveness of PEGDA. These scaffolds for pancreatic *β* cell culture and transplantation create a refined microenvironment that closely mimics physiological conditions for islets, thereby promoting their survival and functionality both in vitro and in vivo (Figure 9A,B).^86^ The strategy of PEG hydrogel encapsulation can not only prolong the survival duration of islet cells after transplantation, but also achieve controlled release of drug molecular/cytokines to promote the repair of damaged parts. Based on this, artificial islets made of PEG loading with small molecules were constructed by Scheiner et al. for controlled VEGF release.^87^ Integrating the angiogenesis function of VEGF, Weaver et al. designed a vascularized synthetic PEG macro‐encapsulation device for islet transplantation (Figure 9C,D).^91^ Furthermore, Coronel et al. formulated an artificial islet based on PEG containing PD‐L1 for the improvement of allograft acceptance and immune modulation.^88^

### 4.2 Islet organoids on the microfluidic chip

The concept of “organ‐on‐a‐chip” has emerged as an innovative platform for scientific investigations. While islets are a small yet crucial part of the human body, they rely on abundant perfusion from the circulatory system. This requirement is met through the innovative use of microfluidic chips. The development of islet‐on‐a‐chip, in addition to multi‐organ‐on‐a‐chip systems that incorporate the islet module, is currently being explored. These models are designed to closely replicate the micro‐physiological environment of natural islets, which leverage advancements in biomaterials to further revolutionize the field of organ‐on‐a‐chip technology.

#### 4.2.1 Islet‐on‐a‐chip system

**FIGURE 10:**
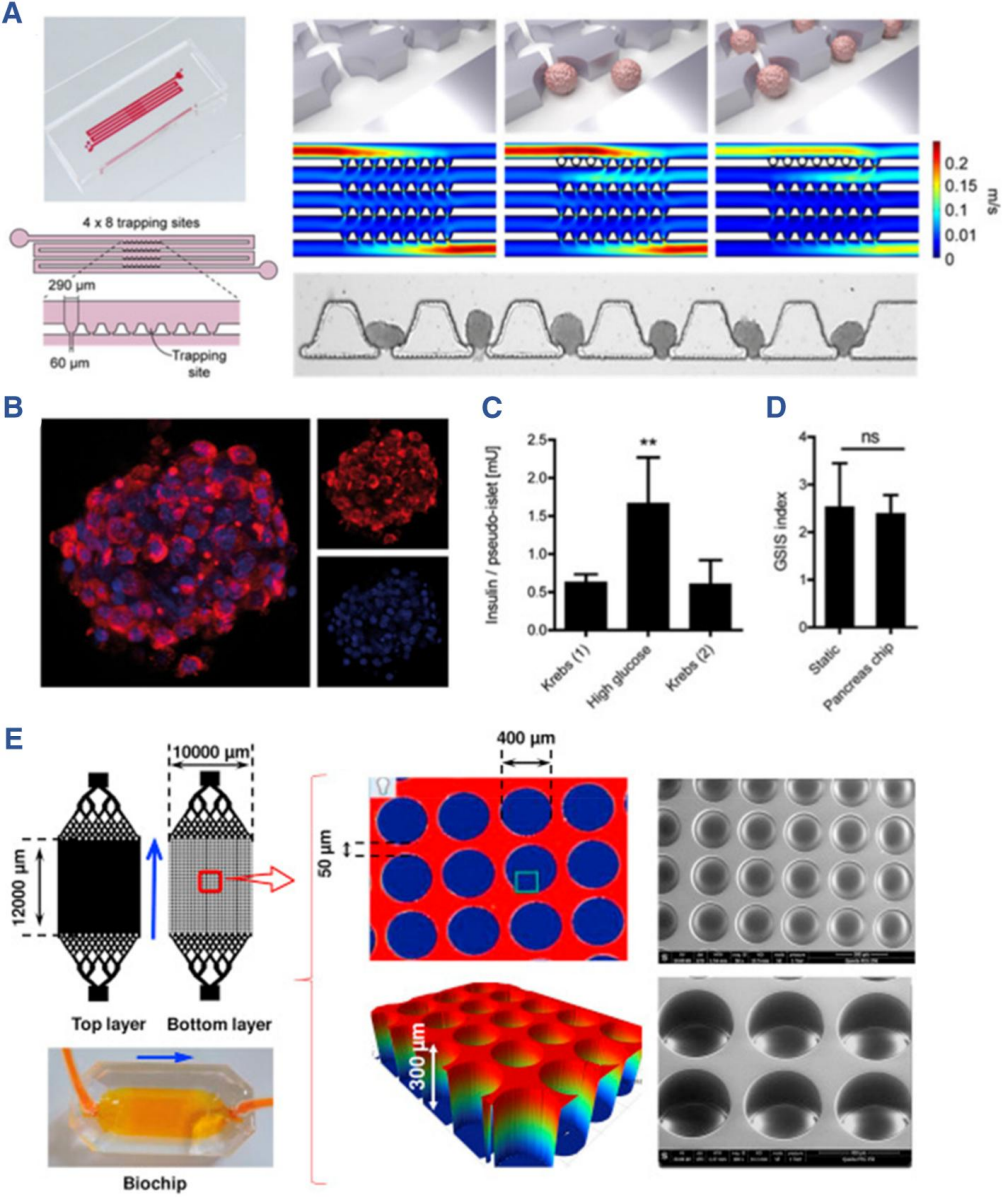
Islet‐on‐a‐chip systems. (A) Non‐invasive marker‐independent high content analysis of a micro‐physiological human pancreas‐on‐a‐chip model. (B) Immunofluorescence images of insulin staining (red). C–D, Insulin secretion (C) and stimulation index (D) under GSIS. *Source*: Reproduced under terms of the CC‐BY license.^92^ Copyright 2020, The Authors, published by Elsevier B.V. (E) Microwell‐based pancreas‐on‐chip model enhances gene expression and functionality of rat islets of Langerhans. *Source*: Reproduced with permission.^93^ Copyright 2020, Elsevier B.V.

Zbinden and their team engineered a human pancreas‐on‐a‐chip that enables non‐invasive real‐time monitoring of homogeneous pseudo‐islets using Raman microscopy. This study involved the cultivation of pseudo‐islets derived from the EndoC‐βH3 cell line on the chip under dynamic perfusion conditions. By utilizing this microfluidic platform, the authors were able to assess the glucose responsiveness and molecular markers of islet organoids (Figure 10A–D).^92^ These findings suggest that the islet‐on‐a‐chip has the potential to advance studies in the realm of organ‐on‐a‐chip technology, extending beyond the field of the pancreas and diabetes. In a separate study, Essaouiba and colleagues developed an islet biochip consisting of two PDMS layers hosting primary rat islets. This microfluidic biochip was designed to enable the long‐term culture of islet organoids while preserving their viability and functionality. Furthermore, the researchers evaluated the responsiveness of islet organoids to glucagon‐like peptide 1(GLP‐1) stimulation, underscoring the superiority of the perfused biochip over conventional petri dishes for islet culture (Figure 10E).^93^

**FIGURE 11:**
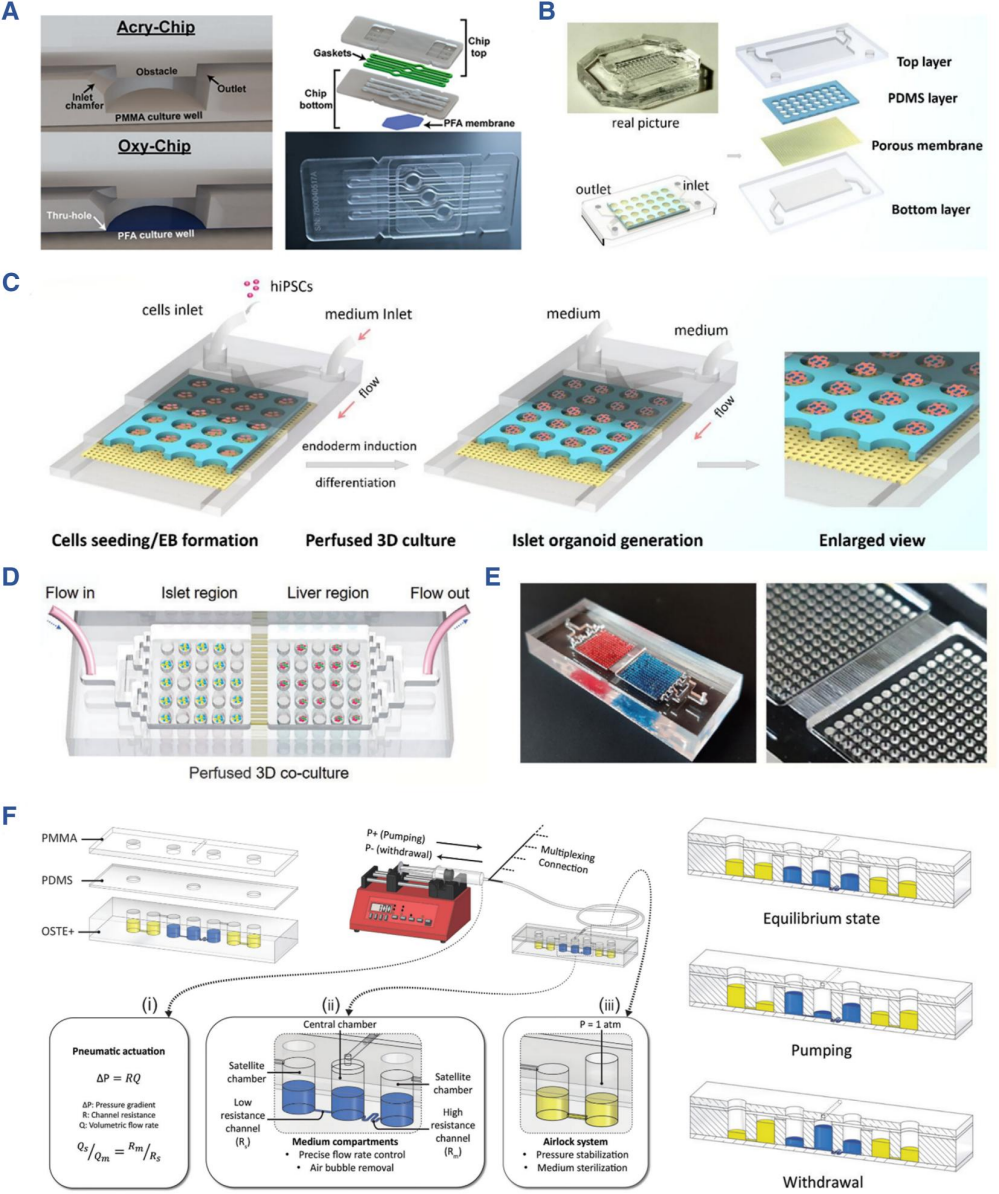
Multifunctional islet‐on‐a‐chip systems. (A) Acry‐Chip and Oxy‐Chip for constructing micro‐physiological systems. *Source*: Reproduced with permission.^20^ Copyright 2021, The Authors, published by American Association for the Advancement of Science. (B) Construction of the islet‐on‐a‐chip. (C) Perfused islet‐on‐a‐chip derived from stem cells. *Source*: Reproduced with permission.^94^ Copyright 2019, Royal Society of Chemistry. (D) Recapitulate liver‐islet axis on a chip. (E) The two‐organ‐on‐a‐chip and the enlarged view. *Source*: Reproduced under terms of the CC‐BY license.^26^ Copyright 2022, The Authors, published by John Wiley and Sons. (F) A pneumatically actuated micro‐physiological device that enables efficient crosstalk between 3D primary human liver cultures and intact human pancreatic islets on a chip. *Source*: Reproduced under terms of the CC‐BY license.^25^ Copyright 2022, The Authors, published by John Wiley and Sons.

To recreate a physiological microenvironment, Patel and their team established a poly (methyl methacrylate) (PMMA) chip known as the Acry‐Chip.^20^ They also introduced a PMMA‐based chip featuring an oxygen‐permeable perfluoroalkoxy (PFA) membrane, referred to as the Oxy‐Chip, suitable for rat and human islet culture. These platforms enabled the achievement of 3D culture of islets, serving the purposes of investigating physiological and pathological mechanisms, real‐time monitoring, and functional evaluation (Figure 11A). In a related endeavor, Tao and their colleagues introduced a dynamic islet culture platform designed to facilitate the differentiation and maturation of heterogeneous human induced pluripotent stem cell (hiPSC)‐derived islets. This innovation demonstrated the proof‐of‐concept for integrating stem cell developmental biology with the islet‐on‐a‐chip (Figure 11B,C).^94^

#### 4.2.2 Pancreas‐liver crosstalk system

Bauer and colleagues introduced a groundbreaking two‐organ‐chip (2‐OC) using replica molding of two PDMS layers, incorporating spatially dependent microwells. To delve into the intricate interactions between islets and the liver, the authors co‐cultured islet and liver organoids with human islets, differentiated HepaRGs, and primary human hepatic stellate cells (HHSteC) for a consecutive 15‐day period on the 2‐OC platform. By comparing these results with traditional monolayer cultures, they convincingly demonstrated the 2‐OC's instant observation, dynamic perfusion, as well as 3D culture of islet organoids, making it a promising candidate for physiological research.^95^

In addition to physiological research, organ‐on‐a‐chip platforms can be used in the construction of pathological models and drug testing. In a study led by Tao et al., the dynamic microfluidic platform served as a stage to recreate the dynamic crosstalk between the liver and pancreatic islets.^26^ HiPSC‐derived liver and islet organoids were used to establish both normal and diabetic models of the liver‐pancreatic islet axis. These models were employed for morphological and functional tests, and notably, metformin, a commonly used diabetes treatment, was introduced as an intervention factor. The results demonstrated metformin's capacity to restore mitochondrial function in the perfused two‐organ chip, offering a valuable tool for reducing reliance on animal models in drug development (Figure 11D,E). This study is an inspiring step toward exploring physiological and pathological states using two‐organoid systems.

More recently, Zandi Shafagh and colleagues have designed an innovative air‐driven microfluidic system to simulate the prediabetic condition by co‐culturing human primary islets and hepatocytes.^25^ The interactions between the islet and liver were compellingly demonstrated by exposing the islets to glucose levels typical of prediabetes. Intriguingly, transcriptomic analyses revealed that the cellular signaling pathways of the islet and liver maintained their independence even under these stimuli (Figure 11F). They have provided a practical platform for two‐organ‐on‐a‐chip systems, ideal for establishing models relevant to precision medicine, and this work is poised to drive further adoption of organ chips in the fields of mechanistic research, drug screening, and regenerative medicine.

**FIGURE 12:**
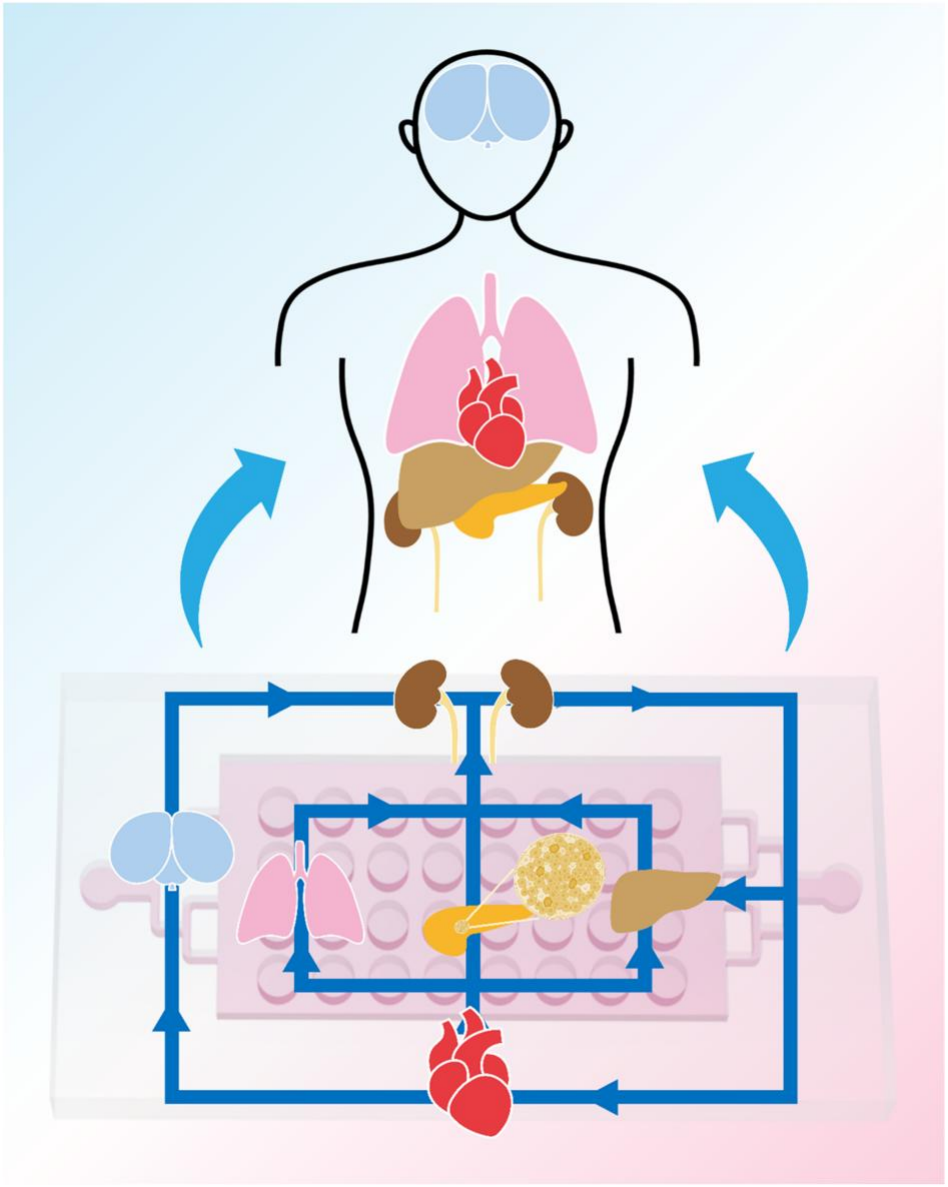
Multi‐organs‐on‐a‐chip system based on the microfluidic chip.

Moreover, multi‐organ‐on‐a‐chip technology enables the in vitro simulation of dynamic interactions between different organs. In order to replicate the functions of human organs and tissues accurately, various organ‐on‐a‐chip models have been developed. These include lung‐on‐a‐chip,^96^ heart‐on‐a‐chip,^97^, ^98^ brain‐on‐a‐chip,^99^, ^100^ liver‐on‐a‐chip,^101^, ^102^ kidney‐on‐a‐chip,^103^ and pancreas‐on‐a‐chip.^104^ Integrating the islet‐on‐a‐chip into the multi‐organ‐on‐a‐chip system is crucial as it regulates systemic endocrine processes that impact the functioning of the human body's organs. The relationships between human organs are highly intricate. In this case, the future research is moving from single‐organ chips to multi‐organ chips to better capture this complexity (Figure 12).

### 4.3 Devices for islet transplantation

**FIGURE 13:**
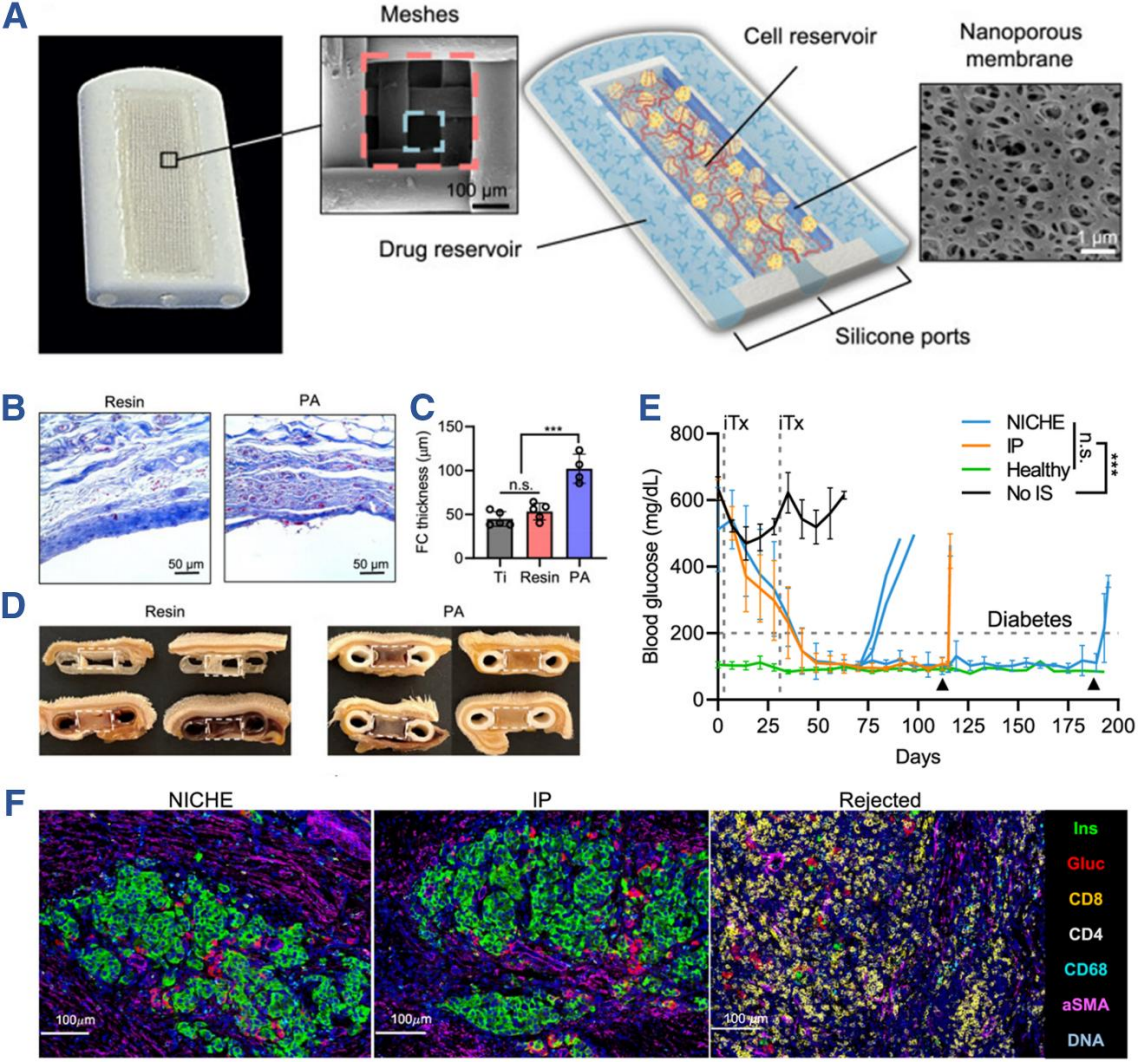
Neovascularized Implantable Cell Homing and Encapsulation (NICHE) for islet allotransplantation. (A) Optical image of NICHE and annotated rendering of NICHE and scanning electron microscopy (SEM) images of the two‐layer mesh and nanoporous membrane. (B) Resin and PA devices implanted in rats for 6 weeks. (C) Quantification of fibrotic capsule thickness around medical grade titanium, resin, and PA. (D) Resin and g PA NICHE implanted subQ for 6 weeks. Dashed lines indicate cell reservoir. (E) BG measurements of diabetic rats transplanted with islets in NICHE cell reservoir receiving local (NICHE) or systemic (IP) immunosuppression, no immunosuppression (No IS), and healthy controls, iTx = islet transplant. Only NICHE and IP rats that achieved euglycemia are plotted. (F) Imaging mass cytometry of cell reservoir tissues from NICHE, IP, and rats with rejected grafts. *Source*: Reproduced under terms of the CC‐BY license.^105^ Copyright 2022, The Authors, published by Springer Nature.

Paez‐Mayorga and their research team introduced an innovative device called the Neovascularized Implantable Cell Homing and Encapsulation (NICHE) for islet allotransplantation.^105^ The NICHE device, roughly the size of a coin, featured dual reservoirs designed for loading cells (referred to as the cell reservoir, or CR) and drugs (known as the drug reservoir, or DR). This device exhibited the capability to facilitate local vascularization, immune suppression, and the fundamental function of insulin secretion. To construct NICHE, they utilized biocompatible polyamide (PA 2200) as the bioprinting material, employing stereolithography 3D printing technology. Specifically, the CR and DR were separated by a two‐layered polyethersulfone (PES) nanoporous membrane, with the ports constructed using implant‐grade silicone adhesive. Upon implanting NICHE into subcutaneous spaces, it resulted in a sustained reduction in blood glucose levels in diabetic rat models over the course of 150 days. Additionally, the transplantation of NICHE into non‐human primates further validated its effectiveness in promoting vascularization and immunosuppression (Figure 13).

## 5 CLINICAL APPLICATIONS

While there remain certain technical challenges to be addressed, the islet organoid technology has proven to be a significant boon for advancing human islet research, particularly in the domains of islet development, diabetes modeling, and islet transplantation. It has been 2 decades since the introduction of the Edmonton protocol, which marked a pivotal moment when cadaveric islets ceased to be the sole source for transplantation.^106^ In recent years, clinical trials aimed at diabetes treatment based on stem cells have been both proposed and conducted.

In 2014, ViaCyte initiated the pioneering trial involving the transplantation of hESC‐derived pancreatic progenitor cells for the treatment of Type 1 Diabetes Mellitus (T1DM) (NCT02239354). Subsequently, in January 2021, Semma Therapeutics in collaboration with Vertex launched an investigational new drug (IND) trial involving hESC‐derived *β* cells for the treatment of T1DM (NCT04786262). Notably, ViaCyte has recently reported interim results from a Phase I/II clinical trial (NCT03163511), wherein human pancreatic endoderm cells were transplanted into macroscopically encapsulated devices to treat T1DM.^107^, ^108^ In this study, 17 patients diagnosed with T1DM were recruited to assess the safety, tolerability, and effectiveness of macro‐encapsulated pancreatic endoderm cells within a year. Encouragingly, the transplanted grafts were observed in the majority of patients without any instances of teratoma formation or severe graft‐related adverse effects. Furthermore, at the 26‐ and 52‐week marks post‐implantation, there was a noticeable increase in C‐peptide and insulin secretion in the recipients, including postprandial C‐peptide secretion. These outcomes provide promising encouragement for the development of human islet organoids. As a result, the thriving field of human islet regeneration is drawing regenerative medicine closer to diabetic patients and is anticipated to play a significant role in addressing the clinical transplantation requirements for cadaveric islets.

## 6 PERSPECTIVES AND CONCLUSION

In the year 2000, the introduction of the Edmonton protocol for clinical islet transplantation marked a turning point, sparking extensive research into artificial islets to enhance diabetes treatment outcomes. This review delves into the design and fabrication of artificial islets, approached from the perspectives of both biology and tissue engineering. Initially, we explore the biological structures and functions of native islets. We then proceed to examine the developmental protocols employed in generating islets from stem cells. Furthermore, we summarize the strategies employed in creating artificial islets, with a particular focus on hydrogels. Finally, we provide a brief overview of the materials and devices utilized in artificial islets for clinical applications. Throughout, our focus is on the synthesis of artificial islets, striving to bridge the realms of developmental biology, clinical medicine, and tissue engineering.

Despite the progress made in the last few decades, challenges remain for artificial islets constructed by developmental and biological strategies. Improvements on the differentiation protocols have enhanced the induction efficiency of SC‐β cells, while the resultant insulin secreting function is still inferior to human islets. To address this issue, single‐cell RNA sequencing can offer inspirations on producing SC‐β cells resembling natural islets. In addition, the off‐target differentiation causes infinite proliferation of undesired cells, even results in tumorigenesis. To eliminate those off‐target cells, cell sorting methods such as fluorescence or magnetic activated strategies can purify the cell types. Moreover, the ethical issues should be taken into consideration as the obtain and applications of stem cells need to be strictly adhered to ethics committee regulations.

Although numerous research for fabricating artificial islets have been investigated based on biomaterials, the clinical practices employing biomedical strategies are still at the primary stage. Firstly, the biomaterials for encapsulating islet cells require the properties of high biocompatibility, appropriate degradability, feasibility, and most importantly, the capacity of supporting islet cells while allowing insulin secretion. The existing strategies for designing artificial islets are able to meet one or several of those requirements. Nevertheless, a universal engineering strategy for artificial islets has not yet been founded. Secondly, the absence of standard criteria for evaluating artificial islet results in products with various qualities. Besides, the outstanding performance of fabricated artificial islets in vitro, even in animal models of rodents and non‐human primates, is unable to predict clinical results of transplantation, which restricts further applications in clinical situations. To achieve positive clinical outcomes, explorations on the transplantation site, the oxygen and nutrient supply, the immune protection, as well as the vascularization of artificial islets are still anticipated.

Despite significant progress made in the last 2 decades, engineers and clinicians continue to grapple with challenges related to artificial islets. Firstly, the limited availability of cell sources poses a significant hurdle for clinical transplantation in diabetes treatment. The scarcity of cadaveric islets is insufficient to meet the growing global demand for transplantation, given the increasing number of diabetes patients. To address this dilemma, stem cells hold promise for generating SC‐islets. However, concerns about tumorigenesis and off‐target cell differentiation hamper the safety of transplantation using stem cells. Secondly, the underlying pathological mechanisms of diabetes, particularly type 1 diabetes, remain incompletely understood, making it a critical factor in artificial islet design. Additionally, immune attacks on transplanted cells by the host's immune system result in cell mass loss, and this challenge can be overcome through cell encapsulation with biomaterials. Nevertheless, the survival of artificial islets is jeopardized by insufficient nutrient and oxygen supply, necessitating advanced modification of biomaterials with tailored chemical components and physical structures. Furthermore, the concept of the islet‐on‐a‐chip serves as an innovative platform for islet research, with a particular focus on the interplay between organs, in line with the broader trend of organs‐on‐a‐chip. Accurately replicating the structures and functions of organs or tissues on microfluidic chips is crucial. Ultimately, quality control considerations are essential for maintaining batch‐to‐batch consistency. While challenges persist, artificial islets hold immense potential in the realm of organoid exploration and diabetes treatment in the future.

## AUTHOR CONTRIBUTIONS

Ling Li, Stephen J. Pandol, and Feika Bian conceived the idea; Jingbo Li wrote the manuscript; and Lingyu Sun revised the manuscript.

## CONFLICT OF INTEREST STATEMENT

The authors declare no competing financial interests.
